# How value‐based healthcare can improve the work of memory clinics

**DOI:** 10.1002/alz.089190

**Published:** 2025-01-09

**Authors:** Anna R. Schönberger, Annika Steinmetz, Ann‐Katrin Schild, Lucrezia Hausner, Gloria Spielmann‐Benson, Lutz Frölich, Frank Jessen

**Affiliations:** ^1^ University Hospital Cologne, Cologne Germany; ^2^ Central Institute of Mental Health, Medical Faculty Mannheim, University of Heidelberg, Mannheim Germany

## Abstract

**Background:**

Value‐based healthcare (VBHC) is a novel concept derived from economic research which is recently implemented in various medical departments and facilities. Additionally to an improvement of patient care, it postulates a reduction of expenses by providing patients with what they really need. Value in this context is defined as outcomes achieved for patients relative to the required costs. In VBHC collecting patient‐reported outcome measures (PROM) and patient‐reported experience measures (PREM) is essential to clarify patients’ needs. According to the VBHC approach, a modification of patient care based on PROM and PREM achieves a higher value and more efficient treatment for patients.

We have implemented the VBHC approach at two German memory clinics to test its applicability in this novel setting. We expect to gain concrete aspects for improving the diagnostic process for patients, relatives, and employees.

**Methods:**

In each memory clinic we interviewed 25 patient‐relative dyads with regard to PREM and PROM. In addition, we conducted an employee survey about current problems and potential suggestions for improvement. Based on these results, respective modifications of the diagnostic process took place in both memory clinics. Thereafter a second evaluation, interviewing another set of 25 dyads was carried out.

**Results:**

In the first evaluation the majority of patients of both memory clinics quoted the length of the diagnostic process as main negative experience. The duration from the first visit to the final diagnosis took several months which was experienced as highly stressful for patients and their relatives. Hence, the diagnostic process was shortened to a duration of approx. 3‐4 weeks. In the second evaluation preliminary data indicate a significantly better experience of patients and family members with the diagnostic process (PREM) after modification. Other aspects reported by patients, relatives as well as employees offer additional perspectives for improvement.

**Conclusions:**

This is the first study implementing the VBHC approach in a memory clinic setting. We found that VBHC can indeed improve patient care and may contribute in establishing a standardized and optimized procedure in the diagnostic processes.

